# Social Identity and Online Support Groups: A Qualitative Study with Family Caregivers

**DOI:** 10.1007/s12529-023-10203-z

**Published:** 2023-08-07

**Authors:** Rosemary Daynes-Kearney, Stephen Gallagher

**Affiliations:** 1 Department of Psychology, Centre for Social Issues Research, Study of Anxiety, Stress and Health Laboratory, University of Limerick, Limerick, Ireland; 2 Health Research Institute, University of Limerick, Castletroy, Limerick, Ireland

**Keywords:** Family caregivers, Online support groups, Social support, Social identity approach, Social isolation, Reflexive thematic analysis

## Abstract

**Background:**

The purpose of this study was to explore whether a sense of collective or shared group identity was developed in one established online social support group for family caregivers and, if so, what did group identification mean for the group members and how was this cultivated in the group.

**Methods:**

Eighteen semi-structured interviews were carried out with members of a family caregiver online support group in Ireland. Inductive and deductive reflexive thematic analysis drawing on the social identity approach (SIA) generated four main themes and several related subthemes.

**Results:**

The first main theme generated was *The group are a Tribe and the Tribe gets it*, with subthemes: *Invisibility as a common experience*, *my Tribe understands I am more than just a carer* and *Just being part of the Tribe can be enough*. The second main theme generated was the *Tribe is a lifeline and life-changing*, with related subthemes: *Our connection relieves experiences of loneliness and social isolation* and *Sharing knowledge, experience and support made me feel visible*. The third and fourth main themes generated were *The leadership of group moderators helped create the Tribe identity* and *Lessons that can be learnt*. These findings are discussed in relation to the social identity approach, social isolation and loneliness.

**Conclusions:**

We concluded that group identity can be cultivated through considered, active and balanced moderation, creating a safe, welcoming and supportive space where family caregivers are able to have fun and seek information, advice and emotional support, which helps relieve loneliness and social isolation.

**Supplementary Information:**

The online version contains supplementary material available at 10.1007/s12529-023-10203-z.

## Introduction

Social support is a key resource for family caregivers, with a health protective benefit consistently reported between social support and physical and mental health benefits [1]. Online support groups are a form of e-support intervention [2] with a primary focus of cultivating social support and alleviating the social isolation that many family caregivers experience [3]. Many organisations now offer social support groups through an online format. However, little is known about why caregivers join, why they stay, how benefits (if there are any) are cultivated or what the barriers or facilitators to these groups are. A key finding from a recent scoping review (Author 1 and Author 2, under review) was that online support groups did relieve social isolation experienced by caregivers, and a common characteristic of importance to the participants of online support groups was that the groups were made up of similar others. Thoits [4] proposed that similar others are more effective at providing elements of social support due to their shared experience, especially where significant other relationships are disrupted by the caregiving role [5].

Social isolation and loneliness are related but different concepts. Social isolation is an objective measure of social connectedness, while loneliness is the perception that the quality and quantity of social relationships are not meeting social needs [6]. Research into both have demonstrated the negative health effects for people in general [6] and for caregivers specifically [7]. Similarly, many studies have examined what types of social support are perceived in online support groups (e.g. [8]) and what health outcomes are produced (e.g. [9]). Our own research with the same caregiver group in this current study found many types of social support were perceived with this online support group (Daynes Kearney & Gallagher, in draft). However, there are myriad theoretical viewpoints on how these elements are created or influenced. For example, some research has identified the stress buffering model [10], the role of social capital [11], while others have observed uncertainty management [12].

The social identity approach (SIA), made of social identity theory [13] and self-cagetorisation theory [14] adapted for health and well-being [15] and sometimes referred to as the “social cure” [16], examines the role of a shared identity of a group and its effects on the health and well-being of individual members [17]. This model proposes that groups should generally be perceived as meaningful and relevant for health-related benefits to be generated [18]. That is, group memberships can build resilience and better health by the enrichment they provide to people’s lives such as social support, belonging and meaning [19].

**Fig. 1:**
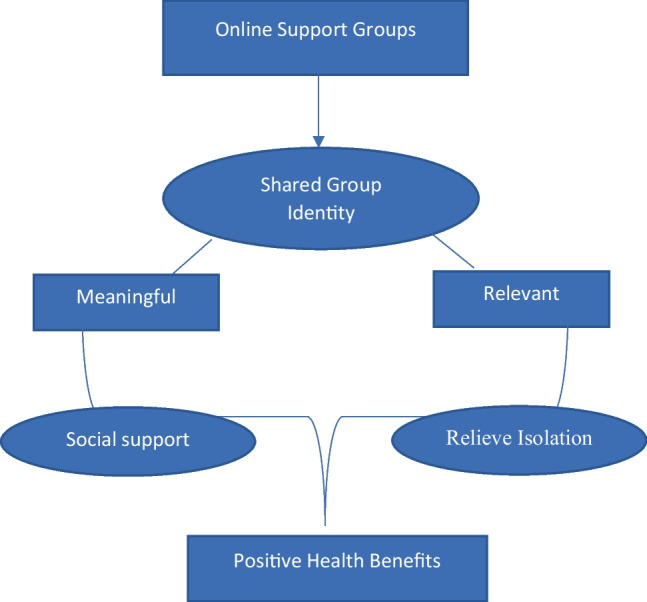
Conceptual model

This theoretical model has been used in research with people with leprosy [20], people with acquired brain injury [18] and recently examining societal COVID-19 responses [21]. Moreover, it has also been employed successfully to gain a better understanding of the health implications of social support groups for people with MS, implying that is may have utility for online support groups for family caregivers [22]. However, to date, there has been no specific research using this approach with family caregivers. This research aims to address this gap in knowledge by exploring why family caregivers feel supported and achieve positive health outcomes, including a reduction in social isolation, in online support groups. As the foundational assumption of SIA is the recognition that individuals can define themselves not only as individuals but also as group members [23], the purpose of this study was to explore whether a sense of collective or shared identity was developed in one established online support groups and, if so, what did it mean for the group members and how was it cultivated in the group (see Fig. 1).

Meaningful and relevant shared group identity in online support groups leads to the development of social support and relieves social isolation contributing to positive health benefits.

## Methodology

### Research Context

Care Alliance Ireland is a network organisation that works with organisations to provide better information and supports to family carers, run initiatives such as National Carers Week and carer specific projects and research (www.carealliance.ie). The Care Alliance Ireland Online Support Group Project, based in Ireland, was established in March 2020 with an online support group for family caregivers. This is run via a private group on Facebook. Within a few months of starting, the group had over 2000 members. As of January 2023, the group has over 5000 members. There was a commitment from the outset that the group would be run by trained moderators drawn from a pool of staff and volunteers who have relevant personal and professional relevant experience. Care Alliance Ireland staff were involved at all stages of the planning and data collection, including setting out their expectations before agreeing to act as gatekeepers to the participants of the group and agreeing a communication schedule for updates once the data collection was completed. The project was also registered on Open Science Framework [OSF Registries | Online Support Groups for Family Caregivers—Qualitative Study]. The project received ethical approval from the institute ethical review board.

### Sample Selection and Data Collection

The sample was self-selecting existing members of this established online support groups. Author 1, in conjunction with the Care Alliance Ireland team, developed several posts and information sheets, introductory posts about the interviewer and goals for the research and running an information session to aid with recruitment. As the interviewer [first author] did not have direct access to the online support groups, these posts were posted into the main feed by the group moderator on several occasions a few weeks apart. Participants could contact the researcher directly by email, and the researcher would respond to answer any questions the person had. Of 32 people who expressed an interest in participating, 18 decided to continue the process to the full interview. The most common reason given for someone not proceeding further was the time commitment required.

We used the model of information power [24] during the recruitment process (see Table 1). Taking the key factors of study aim, sample specificity, established theory, quality of dialogue and analysis strategy into account, we determined that 20 participants was the ideal number, with an option to review if the quality of dialogue was lacking. However, there was a dramatic cultural shift in Ireland in February 2022, when the long-standing social restrictions from the COVID-19 pandemic were lifted. At that stage, we had interviewed 18 participants with a high quality of dialogue and dense specificity of experience. The cultural context of membership of the group during the COVID-19 pandemic lockdowns was a contextual feature of these interviews, and we felt that to continue recruiting in the new cultural landscape could introduce new variables in the members’ experiences, affecting the specificity of information. We also felt that we reached the threshold of information power that would enable an in-depth analysis and took the decision at that time to end recruitment.

**Table 1:** Process to determine sufficient sample size

**Process**	**Decision-making considerations**	**Determination**
Study aim	Narrow or broad	Narrow: focus is specifically on the development of collective identity
Sample specificity	Dense or sparse	Dense: participants would have a spectrum of experiences given the group was more than a year old Participants joined the group at various points during the group life cycle Participants different levels of participation in the group to get a broad range of experiences Focus on quality of interactions as the key factor
Established theory	Applied or not	Applied: Social identity approach
Quality of dialogue	Strong or weak	Strong: Length of interviews averaged 55 min Conversational structure using interview guide where required Reflective notes following interviews identified key areas covered and areas that could require more information
Analysis strategy	Case or cross case	Cross case: Use of inductive and deductive reflexive thematic analysis across entire dataset Reflective notes assessed information gathered to see if adding something new or repetition

In recognition of the demands on caregivers’ time, especially during the lockdown period, where many services such as respite were not fully operational, there were three possible methods of data collection: email questionnaire, online via MS Teams, or Direct Messaging via Facebook Messenger service. Each method was piloted prior to advertisement with former or current family caregivers using a sample interview schedule (see Supplementary Materials A). This interview schedule was refined based on feedback and reflection following these pilot interviews and during the data collection process. The interviewer is a female PhD researcher and has previously worked as a facilitator of face-to-face support groups for family caregivers. She has extensive training and experience in both facilitation and interviewing techniques.

All 18 members chose to be interviewed online by via MS Teams over a period of 4 months (mid-October 2021 to mid-February 2022). All interviews were recorded, and the researcher began each interview with an introduction about the research and her own experience of being a family caregiver and getting consent for the recording and storage of the video interviews. From there, the interviews took the format of a conversation, with the participants’ often speaking for long periods of time with only minimal interruption. The shortest interview lasted 35 min, and the longest was 75 min with an average time of 55 min. The researcher used questions from the interview schedule to cover topics that may not have come up organically in the discussion and at the end to gather demographics (see Table 2). As a mark of appreciation, each participant was sent a €25 one-for-all gift voucher following the interview.

**Table 2:** Demographics of interview participants

**Demographic item**	**Number of participants**
**Gender**	
* Female*	16
* Male*	2
**Age range (mean = 52)**	
* 30–39*	1
* 40–49*	7
* 50–59*	7
* 60–69*	2
* 70–79*	1
**Caring relationship**	
* Parent*	10
* Spouse*	3
* Sibling/in-law*	2
* Child*	2
* More than one caring relationship*	3
**Geographic area**	
* Dublin urban*	3
* Other urban*	4
* Semi-urban/semi-rural*	4
* Rural*	6
**Race/ethic identity**	
* White Irish*	16
* White Other*	2
* Others*	0
**Length of time caring**	
* Under 5 years*	4
* 5–10 years*	1
* 10–15 year*	4
* 15* + *years*	3

### Epistemological and Ontological Stance

The present study was conducted from critical realist stance. The researchers agreed on the viewpoint that objective reality and truth are assumed to exist but that we brought our own life experiences to the research, including as family caregivers, which had a covert and overt influence creating different lenses through which we conducted the process [25]. A reflexive approach allowed us to be aware of our subjectivity [26] and examine our lenses both for biases and strengths that could be brought to the work [27]. A regular schedule of meetings was put in place to facilitate both the discussion of the data and the lenses brought to ensure a coherent interpretation that was rooted in the data [28].

### Data Analysis

Following each interview, Author 1 completed familiarisation notes commenting on the experience of and memorable points from the interview. An advantage of using MS Teams is that it generates an automatic transcript once the meeting is ended. While offered, none of the participants requested a copy of their transcript. This transcript and the video recording were transferred to NVivo (Version 1.7.1(1534): QSR International) and analysed using a process of deductive and inductive reflexive thematic analysis [29]. Reflexive thematic analysis follows six definitive steps [29], and NVivo has been shown to be a helpful tool in this analytic process [30].

Familiarisation with the data was achieved by repeatedly and actively listening to the interviews against the automated transcript and identifying patterns of interest in the data. The video recordings were coded directly, with a comparison against the related automatic transcript. This had the advantage of preserving the context of the discussion, such as tonal inflections, laughter, pauses and observations about body language, which could all be noted using the memo tool. While this reflective process and multiple engagements with the data resulted in the generation of inductive codes, the deductive analysis drew from the social identity approach to direct what to look for in the analysis, such as mutual support, group membership, collectivism, the role of others in the group and the impact of others in the group on the participant (see Table 3). Latent and semantic codes were generated with coding performed manually on a cyclical basis working systematically through the entire dataset.

**Table 3:** Table of final themes and subthemes and how they are relevant to SIA

**Theme name**	**Relevance to SIA**	**Selection of quotes from participants**
**Theme 1:** The group are a Tribe and the Tribe gets it	Categorisation to ingroup — as carers we are a Tribe and other Tribe members understand us. We are different from non-carers	*You feel that you’re isolated from society…but you have a community of your own…I’ve found my Tribe*
Subthemes		
Invisibility as a common experience	We are invisible to others who are not carers	*Everybody knows what everybody else is going through, and the fight with the system…nobody knows about us, we’re kind of just there in the background and…nobody seems to care either…that you’re fighting this battle all the time*
My Tribe understands I am more than just a “carer”	This reflection by my ingroup allows me to both discover the meaning of being a carer and also explore identities that are not being a carer	*It’s remembering that I am a person with a life, with a personality…and caring…really needs to be my secondary role*
Just being part of the Tribe can be enough	Health benefits, such as coping, are felt by identifying with the Tribe even without active engagement. Identification more likely to lead to more engagement	*It’s very supportive, even if you don’t…interact with people, you can read it and you know, and… It just shows that you aren't on your own… there’s others out there in the same circumstances or similar circumstances*
**Theme 2: The Tribe is a lifeline and life-changing**	Identifying with the ingroup has positive benefits such as feelings of belonging, common purpose and support	*I wouldn’t be able to cope as well as I have been coping. That's what gets me through the day and that's what gets me by*
Subthemes		
Our connection relieves loneliness and social isolation	Enhanced social connections, group more likely to be welcoming because of shared connection	*I think it’s nice that people can, even… if it’s only Facebook that they can…actually have somewhere to even just express themselves and say something*
Sharing knowledge, experience and support made me feel visible	Definition of self in relation to the group identity, my behaviour is given a distinct meaning	*It’s amazing how you enter into a particular role that you didn’t necessarily see yourself in. And how one of the positives [is] the skills that I gained being in that caring role*
**Theme 3: The leadership of group moderators helped create the Tribe identity**	Leadership conceived as ability to cultivate group identity Dealing with sub-categorisations in the ingroup — e.g. different caregiving levels	*There are other groups…that I’ve tried and they weren’t as welcoming and as…just make you feel that you can join and…participate in it*
**Theme 4: Lessons that can be learnt**	What can be learnt to develop and maintain group identity	*Not one person said hello to me, which I thought was odd, you know, and they’re all talking to each other…there was no interaction with me at all*

A theme can be defined as “an extended phrase or sentence that identifies what a unit of data is *about* and or what it *means*” [25, p.258]. In the case of reflexive thematic analysis, themes are analytic outputs “actively created by the researcher at the intersection of data, analytic process and subjectivity” and are focused on meaning [26]. Theme generation moved through an iterative process of generating prototype themes to named themes by constructing, deconstructing and reconstructing candidate themes until clarity on the naming of the themes and subthemes was agreed to tell the overall story about the data [31]. This process was iterated during all phases of the research, including the writing of this paper. The CoreQ checklist was completed at the completion of write-up (see Supplementary Material B).

## Findings

The data analysis process resulted in four main themes with associated subthemes, presented below and illustrated using relevant verbatim quotes (see Table 3).

### Theme 1: “The Group Are a Tribe and the Tribe gets It”

A dominant feature across almost all the interviews was the sense of togetherness that participants felt in the group. More than one person referred to the feeling of “all being in the same boat” through shared experiences and recognition that they were not alone in the caring situation, in fact there were thousands of people going through the same as them.

“That’s what I like about the pages, because even though you don't talk to anyone, you get to read people’s experiences and you get to know that you're not the only person paddling. You know there's a whole boat full of us.” [Participant 16]

Running through all these observations was the expression of how the interactions, friendliness and shared experiences led to a feeling of community, and as directly commented in the below quote, the development of a Tribe.

“You feel that you’re isolated from society…but you have a community of your own…I’ve found my Tribe” [Participant 11].

This expression of feeling part of a Tribe was used by several participants in the interviews, and as such, the interviewers chose the term Tribe as an appropriate central component of the theme formation. This sense of Tribe was very personal for the participants and for some was discussed almost with a sense of relief or revelation that there were others like them. For this reason, we constructed the theme from the perspective of ingroup-identity. Drawing of SIA, this is the development of an ingroup/outgroup dynamic, expressed as being different to non-caregivers (see Table 3).

A characteristic of this sense of ingroup-identity was that group members understood the caring experience without it having to be explained, that other members of the Tribe “just got it”.

“And it’s a non-judgmental forum that you don't get at home, or you don’t get from as close to a relative or a partner or a relative or a parent. They just don’t get it.” [Participant 9]

This offered both a sense of relief to participants that they didn’t have to explain or justify themselves, but also highlighted an absence of understanding from those who are significant in their lives, such as family or close friends.

This sense of ingroup-identity was often reflected with the first author, who in her introduction briefly explained her caregiving responsibilities. The first author noticed on several occasions a change in the body language of the participants, who visibly relaxed when they realised that she was also a caregiver. The below example from one interview illustrates this move from “interviewer-participant (outgroup)” to “caregiver-caregiver” (ingroup).

Interviewer: “I care for my son and my husband in primarily emotional, emotional, caring…”

Participant 8:…“I understand that…I’m my partner’s carer and my oldest son’s carer as well so…I get you”.^1^

In reflecting after this interview, the first author noted that this shared experience led to a relationship with the participant establishing quickly and contributed to the quality of the dialogue and depth of the information shared. In an extract from the reflection on Interview 8, the first author wrote:

*I’m profoundly moved by this interview. I felt quite emotional during it…they just really eloquently hit on the areas that really affect carers; social isolation, connection to a group of people who understood, personal touch…the challenge of making connections (a) as older person (b) as carer.*

In holding the space of honest reflexivity, the first author was mindful that she felt a sense of Tribe with the participants as her own experience as a caregiver was validated by the participants and that this point of view would (and has) influenced the analysis process. To consider the depth of this influence, the first author engaged with ongoing discussions with the second author to be able to hold a transparent account of the analysis.

Although there were no direct questions about the social protection and health system in Ireland, it came up organically in many of the interviews. Participants expressed frustration at the struggle that they had to go through to receive state support. This struggle created an environment of fear and demonstrated that their role and contribution as a family carer was not valued in society, leading to a commonly expressed feeling of invisibility.everybody knows what everybody else is going through, and the fight with the system “nobody knows about us, we’re kind of just there in the background and…nobody seems to care either…that you’re fighting this battle all the time.” (Participant 1)

This invisibility was expressed as a gendered experience, with some observing that caring is not valued as it is traditionally regarded as “women’s work” and male carers experiencing invisibility because “it’s not expected for a man to do that”. Again, this is indicative of a subcategorisation of male and female carers within the ingroup. Some participants expressed that even though they were caring that they did not immediately identify as a carer, nor did they want their relationship with their loved one to be defined by being a carer, but by having to engage with the system, this identity was reinforced, making their other identities invisible.

“I’m just my dad’s daughter…I don’t see myself as his full-time carer, which I am on paper.” [Participant 4]

#### Subtheme 2: My Tribe Understands I Am More Than Just a Family Carer

An outcome of membership of the Tribe was the recognition that being a family carer was only part of a person’s life. This recognition was created both by the moderators of the group and by the group interactions themselves. One group member posted a daily positive thought to the group. These thoughts were generally not related to caring. This action was commented on by multiple participants in the interviews as something that reminded them there was someone thinking of them and that there was more to life than caring.

The online group has a variety of online activities such as gardening club, book club, chair yoga and craft club, and this range was appreciated by the group members that it was recognised that as people, they could have hobbies and interests outside of the caring situation.

“It’s remembering that I am a person with a life, with a personality…and caring…really needs to be my secondary role.” [Participant 2]

Another gesture that was appreciated by the group was the gifts they received from Care Alliance Ireland at different points during the year, such as National Carers Week. While these were sometimes related to caring, such as handbooks, often they were not. These gifts again served to reinforce to the group members that they were valued both for their caring role but also as people who deserved a nice treat.

“Somebody has taken the time to put something in an envelope…put your name on it.” [Participant 2]

“You know it might only be bar of chocolate or a book or something, but its [that] somebody says I recognize that you do something more than you know the average parent.” [Participant 3]

#### Subtheme 3: Just Being Part of the Tribe Can Be Enough

From early in the interview process, it was apparent that the sense of community was felt by people regardless of their level of interaction. The participants all had different levels of engagement, from those that posted and commented daily, to some who self-described as “lurkers”.

“I would say 90% of the group probably don't interact on it on a regular basis, but it’s knowing that that there are people out there that are…voicing your concerns, voicing your opinions, you know…it kinda helps you know…It’s nice to know that that somebody knows we're here.” [Participant 16]

Importantly, those more active participants did not see those less-active participants in a negative fashion, and the term “reader” was used in recognition that there were multiple ways for someone to be part in the group.

“It’s very supportive, even if you don’t…interact with people, you can read it and you know, and… It just shows that you aren't on your own… there’s others out there in the same circumstances or similar circumstances”. [Participant 8]

This ability to express the impact of caring on the participant, such as resentment, feelings of tiredness, or annoyance, freely and without negative responses indicated the importance of similar other experiences in the online group and had a positive impact on the lives of the participants.

“You know some days are bad, some days are brilliant…other days you really, really need someone to talk to. And it's learning to open up and to talk and people say God, you found your voice now haven’t you!” [Participant 12]

### Theme 2: The Tribe is Lifeline and Life-Changing

One of the stated objectives of the online support group is to increase the well-being of family carers. We used the social identity approach to consider if the sense of Tribe as an ingroup had positive health benefits (see Table 3). A recurrent reflection that came up in the interviews was that the group was a lifeline for the participants, particularly during the COVID-19 lockdowns.

“I wouldn’t be able to cope as well as I have been coping. That’s what gets me through the day and that’s what gets me by.” [Participant 6]

The benefits of the group were not just felt while the person was interacting with the group but transferred into their daily lives.

“I’m in better form…I have more patience…it definitely gives me more energy…you come off the group and…something will have happened in it that will resonate with you…maybe something hard or something funny that would have happened and it just energises you.” [Participant 11]

One person directly linked their experience in the group to improving their role as a carer.

“Without the support of this group, I would not be able to be as good as a carer and a mum as I am now.” [Participant 12]

#### Subtheme 1: Our Connection Relieved Loneliness and Social Isolation

A common thread running through the interviews was how lonely and isolating the caring situation can be and how the online group was a way to break that experience. Again, the shared lived experience and ability to freely express oneself were important factors to break this isolation, creating a sense of connectedness, a central component of SIA (see Table 3).

“It’s such an emotional and practical support. …many carers unfortunately experience isolation. And it’s a great platform where when you need help, that lived experience, you can identify with the others.” [Participant 5]

“I think it’s nice that people can, even… if it’s only Facebook that they can…actually have somewhere to even just express themselves and say something.” [Participant 4]

For some participants, the people they met on the group felt like friends and their shared experiences helped with getting through the day.

“It’s like having a quick cuppa with a friend without actually meeting them just to see how their day is going on…like a friend of thousands of people, not that you’re going to meet them, it’s nice to know that they’re having the same day as you, and they’ve managed to get through it.” [Participant 9]

#### Subtheme 2: Sharing Knowledge, Experience and Support Made Me Feel Visible

While participants appreciated the facility to ask for support and advice, what also came through was the importance of sharing their own knowledge and experience and the positive impact that this had on their feeling of connectedness with the group. For some participants, they described caring as a role they didn’t chose, but that through the role they had learnt a lot.

“It’s amazing how you enter into a particular role that you didn't necessarily see yourself in. And how one of the positives [is] the skills that I gained being in that caring role.” [Participant 5]

“The action of offering advice both made the participant feel valued for what they know and made them feel good for being able to be there for someone in need. There was a desire to contribute and meaning ascribed by contributing to the group as a member.”

“It’s that support that you can actually give to a stranger and it’s just…nice to be able to say, listen, hang on in there, you know, this will pass.” [Participant 14].

### Theme 3: The Leadership of Group Moderators Helped Create the Tribe Identity

Several of the participants were involved in other online support groups. They reflected that the feeling of this online support group was very different to the other groups and that it was more welcoming, with little negativity towards each other, inappropriate postings and a tone that was gentle, kind and caring.

“There are other groups…that I’ve tried and they weren’t as welcoming and as…just make you feel that you can join and…participate in it.” [Participant 8]

One participant described how they had observed the interactions in the group for months and that the sense of togetherness encouraged them to become more involved in the group.

“So I’ve always been kind of…reserved to post…comment or anything you know. But this this page seems to be...I like the look of it. I like the…people on it and…I like…it’s kind of a camaraderie …it's friendly.” [Participant 13]

Participants commented that the group was well run and that the form of moderation was a strong influence in the group. The moderators take an active role in posting material on the group. Importantly however, this is not just information-based content, but is often something light-hearted and fun. While information provision is considered an important facet of the group, the additional layer of humour was appreciated as participants felt that it recognised the nuances of family caring as not always being doom and gloom and in turn allowed them to use humour in their own posts and comments.

“There are a few things there for the moderators who put up posts, and those are usually very enlightening, very entertaining.” [Participant 16] 

“It’s an outlet for entertainment…it is entertaining some of the posts that they’ve put up…you’d say they’re mad….but I think all carers are a bit mad anyway because you have to be.” [Participant 8]

This sense of fun was carried through to the online groups and activities and was important to participants to relieving some of the pressures of caring while creating connection and giving a mental and emotional break to the carers.

“You don’t have to be great at answering the questions, it’s a bit of fun and you see other people and get to meet other people. Get to know other people.” [Participant 6]

Participants commented on how kind and caring the moderators were and that they treated everyone the same regardless of who they are or their caring situation. Some participants commented on the diversity of caregiving experiences that members were coming from all types of caregiving situations. For most this was a positive, but for some, there was a sense of sub-categorisation, one person described “short-term and lifetime carers” to distinguish those who are caring for a parent or spouse as opposed to a child. Some others felt that their own caring situation was not intense enough compared to some other members for them to be called a carer. However, despite these perceived differences, the sense of group identity was experienced and cultivated by the actions of other members and the group moderators.

Active moderation was an important tool to address the sub-categorisations within the ingroup. Moderators used their role to encourage members to take part in events who wouldn’t otherwise, not wanting to take a place of someone with more perceived carer burden, contacted male carers personally to welcome them to the group and encourage their involvement and from the outset explained that the group recognised that people may not be able to attend activities on a regular basis due to their caring situation.

“The moderators of the group do they treat everyone the same…it doesn’t matter if there are different levels of care…they treat everyone as a carer regardless of the level…It’s back to the non-judgemental thing. You don’t have to attend a coffee morning and if you miss…nobody is going to say anything only, that’s grand, we’ll see you again, or you’re welcome.” [Participant 17]

During the discussions of safety and privacy in the group, participants were unanimous in the need for a group to feel safe for them to engage. On joining the group, participants were made aware of the rules of the group and commented that these rules were reinforced by the actions of the moderators. If something was observed that did not meet the group rules, this was dealt with quickly by the moderators, and by witnessing their actions, members of the group started self-monitoring to deal with issues themselves quickly and respectfully. Importantly, participants felt this set the boundaries of a space that was open, safe but not over moderated.

“They have to be very special people because they have to be able to make sure while allowing the group users and the group members to put up there, be open and honest. I suppose they have to be careful again that nobody is going to be offended or there’s nothing going off, so I think they take a lot of the responsibility and credit for doing that.” [Participant 17]

### Theme 4: Lessons that Can Be Learnt

Overall, participants in the interviews found the group safe and secure and a positive space to engage in. However, one participant did report that they had experienced a data breach where something that they posted in the group was commented to them in real life by another member of the group who knew them personally and that this was a negative experience for them. This breach happened despite all group members needing to sign an online confidentiality agreement prior to joining the group. The first author reported this occurrence to Care Alliance Ireland as part of the general feedback. The project leader expressed surprise that this had happened.

Other participants spoke about finding it difficult to integrate into the activities online, especially if they were new and the others already knew each other.

“Not one person said hello to me, which I thought was odd, you know, and they’re all talking to each other…there was no interaction with me at all.” [Participant 4]

All participants were asked for suggestions and/or recommendations regarding how this could be addressed; these included introductions at the start of the group, using the text box to communicate and direct messaging the group moderators. Again, the first author reported these experiences to Care Alliance Ireland. The moderators have a programme of training in place and make an active effort to be aware of new group members and took this feedback and the feedback on the data breach to integrate into their processes.

While participants generally expressed delight that there was an online group reaching so many people, some were concerned that with the growth of the group, the sense of Tribe might be diluted or lost. These elements did affect the experience of the members and their level of identification with the group and at times reduced their likelihood to take part in the group or indeed remain as a member of the group. When asked, no one really had any idea how to address this challenge of growth balanced with impact, but there was a general consensus that this is a challenge for all online groups.

## Discussion

The aim of this research was to explore whether a sense of shared identity was present in an established online support group in Ireland and, if so, what it meant for the group members and how this was cultivated. The analysis process generated four main themes and several subthemes to offer an explanation of the mechanisms and factors that were important to the group members and that cultivated a sense of collective or group identity. This study drew on the social identity approach (SIA) which states that groups should generally be perceived as meaningful and relevant for health-related benefits to be generated [19]. The themes identified here suggest that online support groups can be effectively understood from a SIA perspective. In particular, we found a common sense of identity, “Tribe” was generated through shared experiences, relief from social isolation and loneliness and reciprocity in sharing information, knowledge and support (see Table 3).

Our findings support similar findings in recent research. The word tribe can give meaning, a sense of purpose and belonging to group members [32]. Connectedness in online support groups has also been found to be qualitatively different from other relationships, characterized by understanding, recognition, fellowship and joy build on shared common experiences [33]. Being a member of the online support groups was found to have positive benefits for the participants of this study, such a coping better, improved mood, reduction in social isolation and being able to be a better carer [34]. The findings echo existing research on “social cure” strand that indicates ways in which group-based processes such as social support contribute to positive health outcomes [16].

Caring has been found to involve the reconstruction of identities with significant others into “carer” and “cared for” [18]. Online support groups can offer a safe space for renegotiation of these identities [5, 35] as well as a space to safely explore caregiver identity as caregivers and as people [2]. The SIA approach indicates that being exposed to the existence of numerous compatible social identities allows a person to draw on more psychological resources to support their health [19]. The navigation of social identities was present in this online support group, with a key benefit being the types of interactions that enabled the person to be seen as more than a caregiver.

Additionally, a study which examined the effect of social identity on well-being in individuals with MS attending a social support group found that the strength of social identity was essential to the health benefits; those who identified more strongly with their peer group reported better mental health compared to those who were less strongly identified [22]. Further exploration on social support mechanisms, social identity and health in these types of online support groups for family caregivers is recommended. The challenge of how to balance growth with impact is another area for further research.

The group was a method of connecting people together, and significantly, here the role of the moderators was an important factor in creating a safe connected space. From SIA, successful leadership depends on how well a shared sense of identity is created, represented and embedded among group members [23, 35]. Trained moderators have been identified as critical in developing peer support groups [34], where counselling training in peer support groups had benefits such as the use of complex techniques like problem solving [36]. Trained volunteers were also found to use a wider variety and more emotion-focused and therapeutic-like cognitive strategies than lay peer supporters [37]. Research has found that moderators can help reduce the risk of bully or maladaptive interactions in online support groups, where one strategy is to provide reminders of the group rules and expectations [38].

Understanding the leadership role of moderators in creating a shared identity in online support groups for family caregivers has not been explored, and so our findings offer new insights in this area. In our study, it seems that the moderators went further than reminding the group of the expectations by actively modelling these in their interactions with the members. This in turn prompted the group members themselves to take leadership roles in promoting group norms and growing the sense of group identity. Our findings suggest that active, considered and balanced moderation which cultivates a welcoming, supportive and fun tone was key leadership factor in this group and further research to explore this would be welcomed.

While this research was not designed to specifically explore social isolation and loneliness, it arose organically in the interviews as prominent features across all the themes and subthemes. Zhang and Fox [39] found that the disclosure of loneliness is not typically explicit in an online support groups and recommends that support providers be aware of masked loneliness in message postings. As we didn’t include the moderators themselves as part of the interview group, future studies examining the strategies moderators use to identify lonely people in the group would be beneficial.

Pohl et al. [40] found that caregivers of a lower age were more isolated than older caregivers. The mean age in their study (*M* = 56.77) is similar to the mean age of this study (*M* = 52) and may indicate a crucial age range for social isolation in caregivers due to the multiple roles many caregivers occupy [41]; however, there appears to be an absence of research looking at social isolation in multi-role or sandwich family caregivers. The known features of online support groups such as availability, flexibility and perceived anonymity (Daynes Kearney & Gallagher, under review; mean that they are a common place for caregivers seeking support. ﻿Family caregivers are at increased risk of social isolation due to the demands of caregiving [40]; however, there is little research about the effects of social isolation on family caregivers. Further research on social isolation and loneliness for family caregivers would be beneficial to add knowledge in this area.

## Limitations

There are several limitations to this study. This study investigated social identity from individual group members’ perspectives; however, a critique of social identity research is that it investigates group level effects from individual group members’ perspectives, rather than at a group level [20]. It is recommended that more studies on social identity approach for health and well-being with family carers explore group level social identity factors. The data collection process took place in the context of ongoing COVID-19 lockdowns in Ireland, which may be affected the level of loneliness and social isolation the participants were feeling. One of the questions in the interview asked participants why they took part in the study, and many of the respondents said that they often took part in research. This indicates that many of the participants were highly motivated research participants and may be more connected in the group and in their offline lives as well and limit the level of transferability of the findings. Additionally, all participants identified as white, as do the researchers. It is important to acknowledge the role of race in structural inequalities including access to resources, and the lack of diversity indicates a possible gap in people who do not identity as white accessing this group. The transferability of the results of this study is therefore limited, and we caution against their blanket applicability to family caregivers of minority groups in Ireland or elsewhere. Finally, this research focused on an online support group on the Facebook platform, which may again limit the transferability of the findings.

## Conclusion

This study suggests that online support groups for family caregivers are spaces where family caregivers can come together and feel connected. Online support groups if poorly understood theoretically could present challenges for practitioners and participants if they are set up and run without an understanding of what participants are looking for, what they receive from the groups and how this benefits them. These findings indicate that the social identity approach is a useful and perhaps central theoretical framework with which to explore how connectedness is formed and why it is important in this context. Our conclusion from this research is that connectedness can be cultivated through considered, active and balanced moderation, creating a safe, welcoming and supportive space where family caregivers are able to have fun and seek information, advice and emotional support, which helps relieve the loneliness and social isolation family caregivers expressed feeling.

### Supplementary Information

Below is the link to the electronic supplementary material.

Supplementary file1 (PDF 481 KB)

Supplementary file2 (PDF 138 KB)

## Data Availability

Raw data (video recordings and associated raw transcripts) for this data are not publicly available to preserve individuals’ privacy under the European General Data Protection Regulation. Transcripts that have been de-identified are currently being prepared for submission to the University of Limerick repository. Updates to the availability of the dataset will be available on https://osf.io/yqp6n/wiki/Dataset/.

## Footnotes

1 In Ireland, the term carer is used rather than caregiver. We use this term in the analysis process to keep the integrity of the source material.
