# Supplementary material for: Social Identity and Online Support Groups: A Qualitative Study with Family Caregivers
Source: Int J Behav Med. 2023 Aug 7;31(3):479–90. doi: 10.1007/s12529-023-10203-z (PMC11106133; doi:10.1007/s12529-023-10203-z)
Supplement: Supplementary file 2 — Supplementary file2 (PDF 138 KB) [file 12529_2023_10203_MOESM2_ESM.pdf]

Interview Guide

A: Welcome and Settling In

Thanks for taking part in the research study. I appreciate your time and interest. Before we begin, as part of the study, I need to collect some general information for statistics. I can ask you for this now or before the end of the interview.

- **Demographic Details** – these details are for aggregated statistics only. This is to help us understand more about the demographics of family carers which is helpful for planning better services

A: Gender: Male/Female/Other/prefer not to say

B: Age range:

- 18-24 years old
- 25-34 years old
- 35-44 years old
- 45-54 years old
- 55-64 years old
- 65-74 years old
- 75 years or older
- Prefer not to say

C: Ethnic background

- How would you describe your ethnic background?

D: How would you describe the location you live?

Dublin urban/Other Urban/Semi-urban/Rural

Review of Transparency and Consent

Previously, I sent you information about this research study and how any data gathered will be used as well a copy of the consent form.

Do you have questions about any of this before we start?

Can I confirm that in line with the consent options on the consent form, that you consent to take part in this research study. Yes/No

And just to make you aware that you can withdraw consent at any time

So I am going to ask a number of questions during the interview. If you don't understand anything please just ask or if you don't want to answer a question, just say so and we will move on. We can also stop the interview at any time if you decide that you don't want to continue. Is that ok?

Q: I am interested to hear about your understanding of what this study is about…

What drew you to take part in this study?

That's great. (Clarify and or reflect back).

B: Caring Background

1. Tell me about your experience of being a family carer?
2. Would you always have considered yourself a family carer?
3. When did you realise that you were a family carer? What was that like for you?
4. Did someone tell you that you were a family carer? What was that like for you?

C: Becoming a member of the Online Support Group

1. How did you become a member of the CAI Facebook group?
2. How did you find out about the group?
3. Tell me about your first experience of the group?
4. Why did you continue to engage with the group?

D: Engagement with OSG

1. Tell me about how the OSG works once you join?
2. How do you engage with the group?
3. How often do you engage with the group?
4. What is it about the group that you like?
5. Tell me about some of the benefits of being a member of the group?
6. How safe do you feel in this online support group?
7. What impact, if any, has the moderation of the group had on your engagement?
8. If the group closed tomorrow, how would that impact you?

E: Social Identity Measurements

1. Do you feel yourself as a member of this Facebook group?
2. Do you identify with other carers within the group?
3. How has that changed over time – become stronger, weaker….?
4. How would you describe your relationships to others in the CAI OSG?

5. Are you a member of other OSG?
6. If yes, how would you compare your relationships in this OSG to the other OSGs?

F: Impact of Covid

1. The last year has been very strange and very difficult in many ways.
2. Can you tell me about how the Covid pandemic has impacted you?
3. How did you adapt to circumstances? How many times have you had to adapt? What has that been like for you?
4. What strengths and resources do you use?
5. What motivates you to keep going?

G: Technology/Platform/Moderation

1. Tell me about the value of having a support group that is available through an online platform?
2. What is it like having an Online Support Group on Facebook?
3. If the group was moved to a different platform (e.g. a new bespoke platform for online support groups), how would that impact you?

H: Recommendations:

1. If you were to go back to when you decided to join the group, would you still make the decision to join again?
2. Have you any recommendations for CAI re the group?
3. Have you any recommendations for new members about the group?
4. Is there anything that I haven't considered that you think is important that you would like to include now.

Ending

Thank you for taking part in this interview. I really appreciate your time and you sharing your experience and knowledge with me. Before we finish, we will be interviewing people again in around 12 months time so that we can understand how online support groups work over time. Would you be interested in being contacted about this? This doesn't mean that you have to take part in the study.

Longitudinal study Consent: Yes/No
